# Balloon-Expandable Vs Self-Expanding Valves for Transcatheter Aortic Valve Implantation in Internally Stented Surgical Valves

**DOI:** 10.1016/j.cjco.2026.06.013

**Published:** 2026-06-29

**Authors:** Daisuke Sato, Noriaki Moriyama, Yoichi Sugiyama, Hirokazu Miyashita, Tomoki Ochiai, Koki Shishido, Futoshi Yamanaka, Tommi Vähäsilta, Teemu Laakso, Sebastian Dahlbacka, Tiina Vainikka, Juho Viikilä, Shigeru Saito, Mika Laine, Mikko Jalanko

**Affiliations:** a Department of Cardiology, Heart and Lung Center, Helsinki University Hospital and University of Helsinki, Helsinki, Finland; b Department of Cardiology, Shonan Kamakura General Hospital, Kanagawa, Japan; c Department of Cardiovascular Surgery, Heart and Lung Center, Helsinki University Hospital and University of Helsinki, Helsinki, Finland

**Keywords:** transcatheter aortic valve implantation, internally stented valves, Mitroflow, Trifecta, balloon-expandable valve, self-expandable valve

## Abstract

**Background:**

Internally stented (IS) surgical valves, such as Mitroflow (Sorin Group, Arvada, CO) and Trifecta (St. Jude Medical), are known to carry an elevated risk for coronary artery obstruction during valve-in-valve (ViV) transcatheter aortic valve implantation (TAVI) procedures. The data from comparison between balloon-expandable valves (BEVs) and supra-annular self-expanding valves (SEVs) for ViV-TAVI within the IS valve remains unclear. This study aimed to evaluate clinical and hemodynamic outcomes of using BEVs and SEVs for TAVI within an IS valve.

**Methods:**

Between January 2016 and February 2024, propensity score–matching was performed between BEV and SEV groups in patients undergoing ViV-TAVI within an IS valve. The data of baseline characteristics, hemodynamic parameters, and clinical outcomes were retrospectively collected. Primary endpoints were 30-day and 2-year clinical outcomes, including all-cause death, hospitalization for heart failure, and coronary obstruction.

**Results:**

In total, 113 patients were included in this analysis. A total of 63 patients (55.8%) underwent BEV implantation, and 50 patients (44.2%) underwent SEV implantation. Overall, 37 pairs were identified after propensity-score matching. At day 30, the clinical and hemodynamic outcomes were similar between the groups. Patients with an SEV had better post-procedural mean gradient at 1 year compared with those with a BEV (11.7 ± 4.3 mm Hg vs 22.7 ± 8.4 mm Hg; *P* < 0.001). No significant difference was observed between SEVs and BEVs in the cumulative 2-year composite endpoint (log-rank *P* = 0.489).

**Conclusions:**

In patients who underwent ViV within an IS valve, the early- and midterm clinical outcomes were comparable between BEVs and SEVs. However, use of an SEV was associated with superior hemodynamics at 1-year follow-up, vs a BEV.

## Central Illustration

**Figure:**
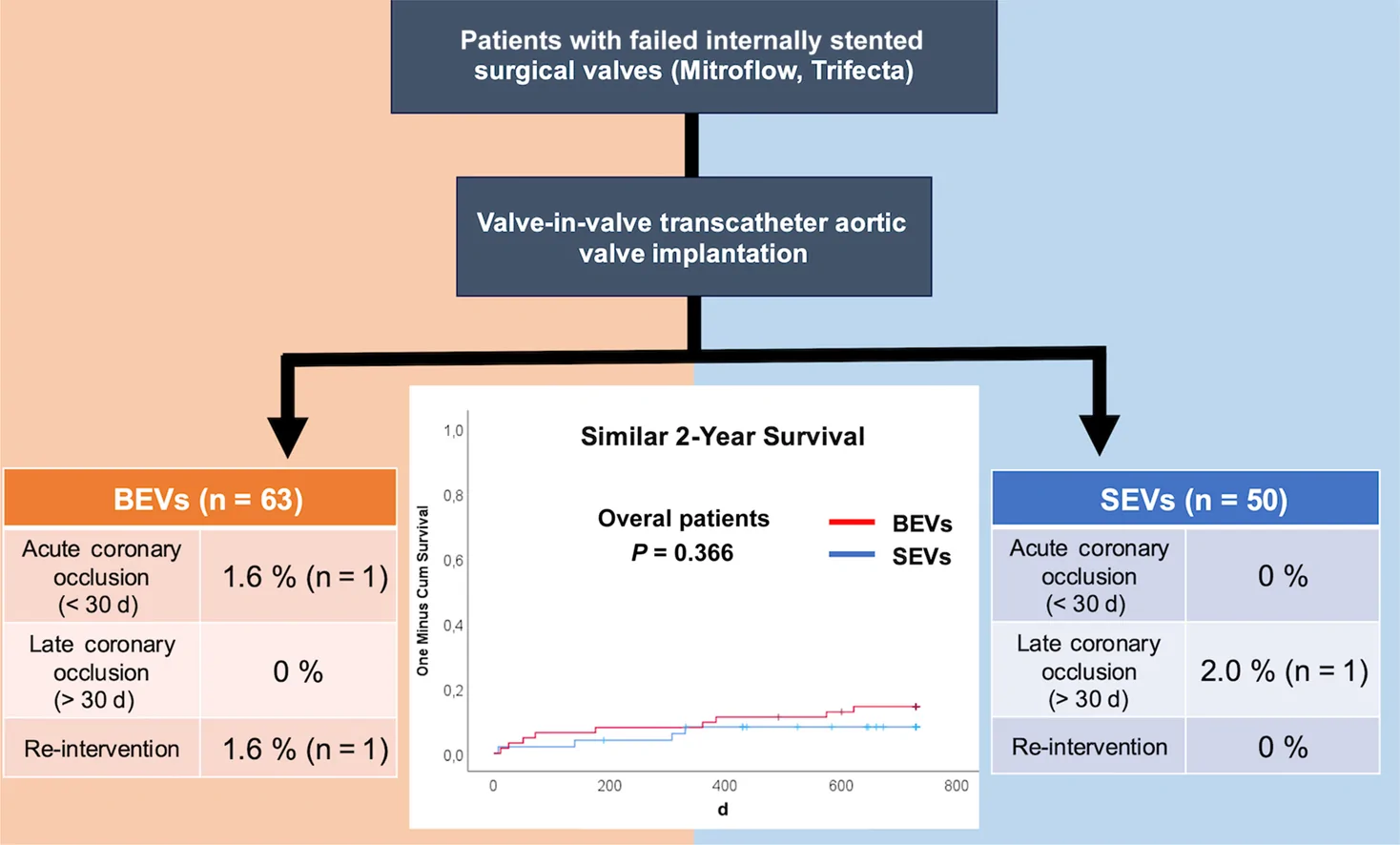

## Résumé

**Contexte:**

Les bioprothèses valvulaires avec armature (BVA), telles que Mitroflow et Trifecta, sont connues pour présenter un risque accru d’obstruction des artères coronaires lors des procédures d’implantation valvulaire aortique par cathéter (IVAC) de type « valve-in-valve » (ViV). Les données comparatives entre les valves expansibles par ballonnet (VEB) et les valves auto-expansibles (SEV) supra-annulaires pour l’IVAC de type ViV avec armature interne restent floues. Cette étude visait à évaluer les résultats cliniques et hémodynamiques des valves BEV et SEV pour l’IVAC avec armature interne.

**Méthodologie:**

Entre janvier 2016 et février 2024, un appariement par score de propension (PSM) a été réalisé entre les groupes BEV et SEV chez les patients ayant subi une IVAC de type « valve-in-valve » avec BVA. Les données relatives aux caractéristiques initiales, aux paramètres hémodynamiques et aux résultats cliniques ont été recueillies de manière rétrospective. Les critères d’évaluation principaux étaient les résultats cliniques à 30 jours et à 2 ans, comprenant la mortalité toutes causes confondues, l’hospitalisation pour insuffisance cardiaque et l’obstruction coronarienne.

**Résultats:**

Au total, 113 patients ont été inclus dans cette analyse. Soixante-trois patients (55,8 %) ont reçu une BEV, et 50 patients (44,2 %) une SEV. Au total, 37 paires ont été constituées après PSM. Au jour 30, les résultats cliniques et hémodynamiques étaient comparables entre les groupes. Les patients ayant reçu une SEV présentaient un gradient moyen post-intervention meilleur à 1 an que ceux ayant reçu une BEV (11,7 ± 4,3 mmHg contre 22,7 ± 8,4 mmHg; *p* < 0,001). Aucune différence significative n’a été observée entre la SEV et la BEV concernant le critère d’évaluation composite cumulé à 2 ans (test logarithmique par rangs : *p* = 0,489).

**Conclusions:**

Chez les patients ayant bénéficié d’une ViV avec une BVA, les résultats cliniques à court et moyen terme étaient comparables entre la BEV et la SEV. Cependant, la SEV était associée à une hémodynamique supérieure à 1 an de suivi par rapport à la BEV.

Valve-in-valve (ViV) transcatheter aortic valve implantation (TAVI) is a well established alternative treatment for patients with degenerated surgical bioprostheses who are at high surgical risk for redo surgical aortic valve replacement.^1^^,^^2^ Among these surgical aortic valves, internally stented (IS) valves (Mitroflow [Sorin Group, Arvada, CO] and Trifecta [St. Jude Medical, St. Paul, MN]) have unique features, in which pericardial leaflets are mounted externally to the valve stent. During deployment of the TAVI prosthesis, these externally mounted pericardial leaflets may be displaced away from the valve stent, potentially leading to coronary obstruction.^3^^,^^4^ In addition, IS valves increase the risk of significant residual stenosis during subsequent ViV-TAVI procedures in patients with small native annuli.^5^ These factors raise concerns about adverse outcomes and long-term durability in patients undergoing ViV-TAVI.

The presence of a small aortic annulus is associated with poor outcomes and suboptimal hemodynamics after surgical aortic valve replacement, which can lead to patient–prosthesis mismatch (PPM) and high-residual gradient.^2^ Previous studies have shown that in patients with failed small surgical aortic bioprostheses undergoing ViV-TAVI, supra-annular self-expanding transcatheter heart valves (SEVs) are associated with a lower transvalvular gradient and better valve performance compared with balloon-expandable transcatheter heart valves (BEVs), without a corresponding increase in adverse outcomes.^2^^,^^6^ However, no studies have directly compared the clinical and hemodynamic outcomes of BEV vs SEV of ViV-TAVI with IS valves. Therefore, this study aimed to compare the clinical and hemodynamic outcomes of BEVs vs SEVs in patients undergoing ViV-TAVI for IS surgical bioprostheses using a propensity score–matched cohort.

## Methods

### Study population

The international TAVI registry (trial identifier: UMIN000040413) comprises retrospectively and prospectively collected data from consecutive patients who underwent TAVI for severe aortic stenosis (AS) or degenerated surgical aortic valves at 2 high-volume centres—Helsinki University Hospital in Finland, and Shonan Kamakura General Hospital in Japan. Between January 2016 and February 2024, a total of 163 patients who underwent ViV-TAVI with an SEV or BEV were enrolled in this analysis. Inclusion criteria were the use of commonly used SEVs and BEVs. The SEVs available during the study period were the Evolut R, PRO, PRO+, and FX (Medtronic, Minneapolis, MN), and the BEVs were SAPIEN 3 and Ultra (Edwards Lifesciences, Irvine, CA). The selection of a BEV or SEV in the present study was influenced by institutional device availability during the study period. Helsinki University Hospital in Finland had access to both BEV and SEV platforms, whereas Shonan Kamakura General Hospital in Japan exclusively used SEVs. We excluded TAVI cases for patients who underwent TAV-in-TAV or TAV-in-without IS. Indications for ViV procedures were categorized according to the mechanism of aortic prosthesis dysfunction as follows: (i) prosthesis stenosis—defined as a mean transprosthesis gradient ≥ 40 mm Hg (severe AS) and aortic insufficiency (AI) less than moderate; (ii) AI: moderate or greater transvalvular AI and a mean transprosthesis gradient < 40 mm Hg; and (iii) mixed—a mean transprosthesis gradient ≥ 40 mm Hg and a moderate or greater transvalvular AI. The prospective registry was established in accordance with the principles of the Declaration of Helsinki and was approved by the institutional clinical research and ethics committee. Informed patient consent was waived due to the retrospective nature of the study.

### Device description and definition

The decision to perform ViV-TAVI was reached by consensus within the multidisciplinary heart team, which included experienced clinical and interventional cardiologists as well as cardiovascular surgeons. At Helsinki University Hospital, the choice between a BEV and an SEV was made through heart team discussion based on patient-specific anatomic and clinical considerations, including the inner diameter of the surgical prosthesis, the risk of coronary obstruction, and patient comorbidities. Transcatheter heart valve (THV) sizing was guided by data from smartphone application-based sizing data, manufacturer charts, 3-dimensional multidetector computed tomography (CT)-based annular measurements, and expert consensus.^6^ Valve sizes of the surgical valves were defined according to the true internal diameter (ID).^7^ Following the procedure, the patients underwent transthoracic echocardiography (TTE) during hospitalization to assess postprocedural hemodynamics and cardiac function. Clinical outcomes and procedural complications, such as stroke, bleeding, vascular complications, acute kidney injury, and newly required pacemaker implantation, were analyzed according to the Valve Academic Research Consortium 3 criteria.^8^

### Coronary protection and risk for occlusion

Coronary protection was performed in cases with a potential risk of coronary obstruction, as determined according to established criteria (virtual valve-to-coronary distance ≤ 4 mm, height of the coronary ostium ≤ 10 mm, and shallow sinus of Valsalva average diameter ≤ 30 mm).^9^ The protection procedure also was performed in cases of specific patient anatomy (presence of leaflet calcium or THV type to be used [BEV vs SEV]). For high-risk features of coronary obstruction, coronary protection was performed before deploying the valve by engaging the coronary ostium with an appropriately sized stent.

### Data collection and outcomes

Baseline clinical, procedural, and follow-up data were prospectively entered into institutional databases maintained at each participating centre. Adverse events were systematically recorded and adjudicated by an independent clinical event committee. All data were derived from a collaborative registry database. Clinical follow-up data were obtained through outpatient visits, review of medical records, or structured telephone interviews at 1 month, 1 year, and annually thereafter. Echocardiographic outcomes at 30-day and 1-year follow-up assessments were analyzed in patients with available follow-up imaging. However, not all patients underwent echocardiography at every scheduled time point, due to death, transfer to other institutions, comorbidities limiting outpatient visits, or patient-related factors. For echocardiographic parameters, only available data were included in the analysis, without imputation.

The primary endpoints were 30-day and 2-year clinical outcomes, including all-cause mortality, hospitalization for heart failure, and coronary obstruction.

### Statistical analysis

Categorical variables were described as absolute numbers with relative percentages and compared using the χ^2^ or Fisher’s exact test, as appropriate. Continuous variables were expressed as mean ± standard deviation (SD) or median (interquartile range [IQR]) and compared using the Student *t*-test or the Mann–Whitney U test, depending on their distribution. To account for baseline differences and selection bias between the SEV and the BEV groups, propensity score–matching (PSM) analysis was performed to create comparable cohorts. The PSM was performed on the basis of a logistic regression model that included the following variables: age, sex, body mass index, Society of Thoracic Surgeons mortality score, hypertension, diabetes mellitus, atrial fibrillation, chronic kidney disease, left ventricular ejection fraction, chronic pulmonary disease, peripheral artery disease, and true ID. Variables were selected based on clinical relevance and prior evidence demonstrating their association with mortality, hemodynamic performance, and the choice of THV type in ViV-TAVI . Patients were matched using the PSM logit with 1:1 optimal matching and a caliper width of 0.2. The cumulative incidence of clinical events was estimated using the Kaplan-Meier method, and differences were assessed using the log-rank test. To address the impact of incomplete echocardiographic follow-up data and leverage the larger sample size, a sensitivity analysis was performed using multivariable Cox proportional hazards regression in the entire unmatched cohort. Multivariate Cox proportional hazards regression analysis was performed to identify independent predictors of the composite endpoints, including all-cause mortality, hospitalization for heart failure, and coronary obstruction at 2 years. Clinically relevant baseline and procedural variables were used with *P*-values < 0.20 in the univariate analysis. A 2-sided *P*-value < 0.05 was considered statistically significant for all analyses. All analyses were carried out using SPSS version 29 (IBM, Armonk, NY).

## Results

### Baseline patient characteristics

During the study period, a total of 113 patients who underwent ViV-TAVI for IS were included in this study. A total of 63 patients were treated with a BEV, and 50 patients were treated with an SEV. The baseline characteristics of the study cohort are shown in Table 1. The SEV group included more female patients, compared with the BEV group (72.0% vs 49.2%, *P* = 0.014). The BEV group had a higher proportion of chronic pulmonary disease and peripheral artery disease than the SEV group (*P* < 0.01 and *P* = 0.011, respectively). Prior percutaneous coronary intervention and coronary artery bypass grafting did not differ between the 2 groups. The median time between the index surgical aortic valve replacement and the ViV-TAVI procedure was similar in both groups. The SEV group had a higher proportion of aortic stenosis than the BEV group (*P* = 0.035). Thereafter, a 1:1 PSM analysis was conducted, resulting in 2 paired groups—SEV (n = 36) and BEV (n = 36) groups (Fig. 1). After the matching, the absolute standardized mean difference (SMD) of all covariates was < 0.2. The patients’ baseline characteristics were well balanced between the BEV and SEV groups, including TTE and multidetector computed tomography variables.

**Table 1:** Baseline characteristics

	Unmatched cohort				Matched cohort			
	BEV (n = 63)	SEV (n = 50)	SMD	*P*	BEV (n = 36)	SEV (n = 36)	SMD	*P*
**Baseline characteristics**								
Age, Y	76.8 ± 6.3	79.3 ± 5.2	0.43	0.237	77.4 ± 6.2	77.8 ± 4.9	0.07	0.228
Female	31 (49.2)	36 (72.0)	0.02	**0.014**	20 (55.5)	22 (61.1)	0.03	0.633
BMI,∗ kg/m^2^	26.1 ± 5.0	24.1 ± 4.6	0.42	0.633	25.0 ± 4.4	25.8 ± 4.1	0.19	0.963
BSA,∗ m^2^	1.8 ± 0.3	1.7 ± 0.2	0.39	0.330	1.76 ± 0.21	1.78 ± 0.17	0.10	0.456
STS score, %	5.4 ± 2.6	6.6 ± 4.4	0.33	0.802	5.44 ± 3.68	5.10 ± 3.88	0.09	0.991
Preoperative NYHA class Ⅲ, Ⅳ	53 (84.1)	39 (78.0)	0.06	0.406	30 (83.3)	30 (83.3)	0.00	1.000
Hypertension	57 (90.5)	43 (86.0)	0.06	0.459	31 (86.1)	32 (88.9)	0.01	1.000
Diabetes mellitus	20 (31.7)	11 (22.0)	0.04	0.249	10 (27.8)	10 (27.8)	0.00	1.000
Renal failure†	33 (52.4)	33 (66.0)	0.00	0.145	18 (50.0)	20 (55.9)	0.03	0.637
Chronic lung disease	17 (27.0)	2 (4.0)	0.07	**<0.001**	3 (8.3)	2 (5.6)	0.01	1.000
Prior MI∗	9 (14.3)	3 (6.3)	0.03	0.177	4 (11.1)	3 (8.8)	0.01	1.000
Prior PCI∗	11 (17.5)	3 (21.4)	0.04	0.087	5 (13.9)	3 (8.3)	0.03	0.710
Prior CABG	23 (36.5)	12 (24.0)	0.05	0.153	13 (36.1)	9 (25.0)	0.06	0.306
Prior PPI	7 (11.1)	3 (6.0)	0.02	0.508	3 (8.3)	2 (5.6)	0.01	1.000
PAD	13 (20.6)	2 (4.0)	0.05	**0.011**	3 (8.3)	2 (5.6)	0.01	1.000
Atrial fibrillation	25 (39.0)	20 (40.0)	0.02	0.973	14 (38.9)	16 (44.4)	0.03	0.633
Prior stroke	9 (14.3)	4 (8.0)	0.02	0.381	5 (13.9)	4 (11.1)	0.01	1.000
Time from SAVR to TAVI, Y	8.7 ± 2.5	8.3 ± 2.8	0.15	0.205	8.9 ± 2.9	9.2 ± 2.4	0.11	0.155
**Echocardiographic data**								
Preoperative LVEF,∗ %	54.6 ± 13.1	56.5 ± 13.0	0.15	0.870	55.6 ± 11.0	55.7 ± 11.8	0.01	0.754
AV max,∗ m/s	3.9 ± 0.6	3.9 ± 0.7	0.00	0.254	3.5 ± 0.8	4.0 ± 0.7	0.66	0.349
Pre-mean gradient∗	37.4 ± 13.9	37.5 ± 15.4	0.01	0.959	36.1 ± 14.9	39.3 ± 16.4	0.20	0.670
Aortic valve area,∗ cm2	0.81 ± 0.30	0.77 ± 0.24	0.15	0.879	0.75 ± 0.23	0.77 ± 0.24	0.09	0.575
**CT data**								
STJ height, mm	21.1 ± 5.8	19.4 ± 6.1	0.29	0.533	20.2 ± 5.1	20.2 ± 5.6	0.00	0.262
Right coronary height, mm	14.8 ± 4.2	11.7 ± 4.5	0.88	0.840	14.3 ± 3.7	12.3 ± 4.5	0.48	0.761
Left coronary height, mm	10.8 ± 3.8	9.4 ± 4.0	0.36	0.875	10.4 ± 3.2	10.1 ± 4.2	0.08	0.669
VTC-RCA, mm	6.0 ± 3.3	6.3 ± 2.6	0.10	0.683	5.1 ± 2.2	6.8 ± 2.8	0.67	0.854
VTC-LCA, mm	5.2 ± 3.0	6.1 ± 2.2	0.34	0.584	4.9 ± 2.7	6.1 ± 2.0	0.50	0.799
**Valve pathology**								
AS	24 (38.1)	29 (58.0)	0.02	**0.035**	12 (33.3)	23 (63.9)	0.15	**0.009**
AI	18 (28.6)	12 (24.0)	0.03	0.585	13 (36.1)	7 (19.4)	0.08	0.114
Mixed	21 (33.3)	9 (18.0)	0.05	0.067	11 (30.6)	6 (16.7)	0.07	0.165
True valve ID, mm	18.7 ± 1.5	18.5 ± 1.4	0.14	0.696	19.1 ± 1.7	18.9 ± 1.8	0.11	0.987
Small index device (true ID < 19 mm)	44 (69.8)	42 (84.0)	0.01	0.080	27 (75.0)	28 (77.8)	0.01	0.781

Values are expressed as total number (percentage), mean ± standard deviation, or median (interquartile range). Boldface indicates significance.

AI, aortic insufficiency; AS, aortic stenosis; AV, aortic valve; AVmax, aortic valve maximum blood flow velocity; BEV, balloon-expandable valve; BMI, body mass index; BSA, body surface area; CABG, coronary artery bypass grafting; CT, computed tomography; ID, internal diameter; LCA, left coronary artery; LVEF, left ventricular ejection fraction; MI, myocardial infarction; NYHA, New York Heart Association; PAD, peripheral artery disease; PCI, percutaneous coronary intervention; PPI, permanent pacemaker implantation; RCA, right coronary artery; SAVR, surgical aortic valve replacement; SEV, self-expanding valve; SMD, standardized mean difference; STJ, sinotublar junction; STS, Society of Thoracic Surgeons; TAVI, transcatheter aortic valve implantation; VTC, valve-to-coronary distance.

∗ Missing data: BMI, n = 1; BSA, n = 1; prior MI, n = 2; prior PCI, n = 2; preoperative LVEF, n = 1; AVmax, n = 2; pre-mean gradient, n = 3; aortic valve area, n = 21.

† Estimated glomerular filtration rate < 60 mL/min per 1.73 m^2^.

**Figure 1:**
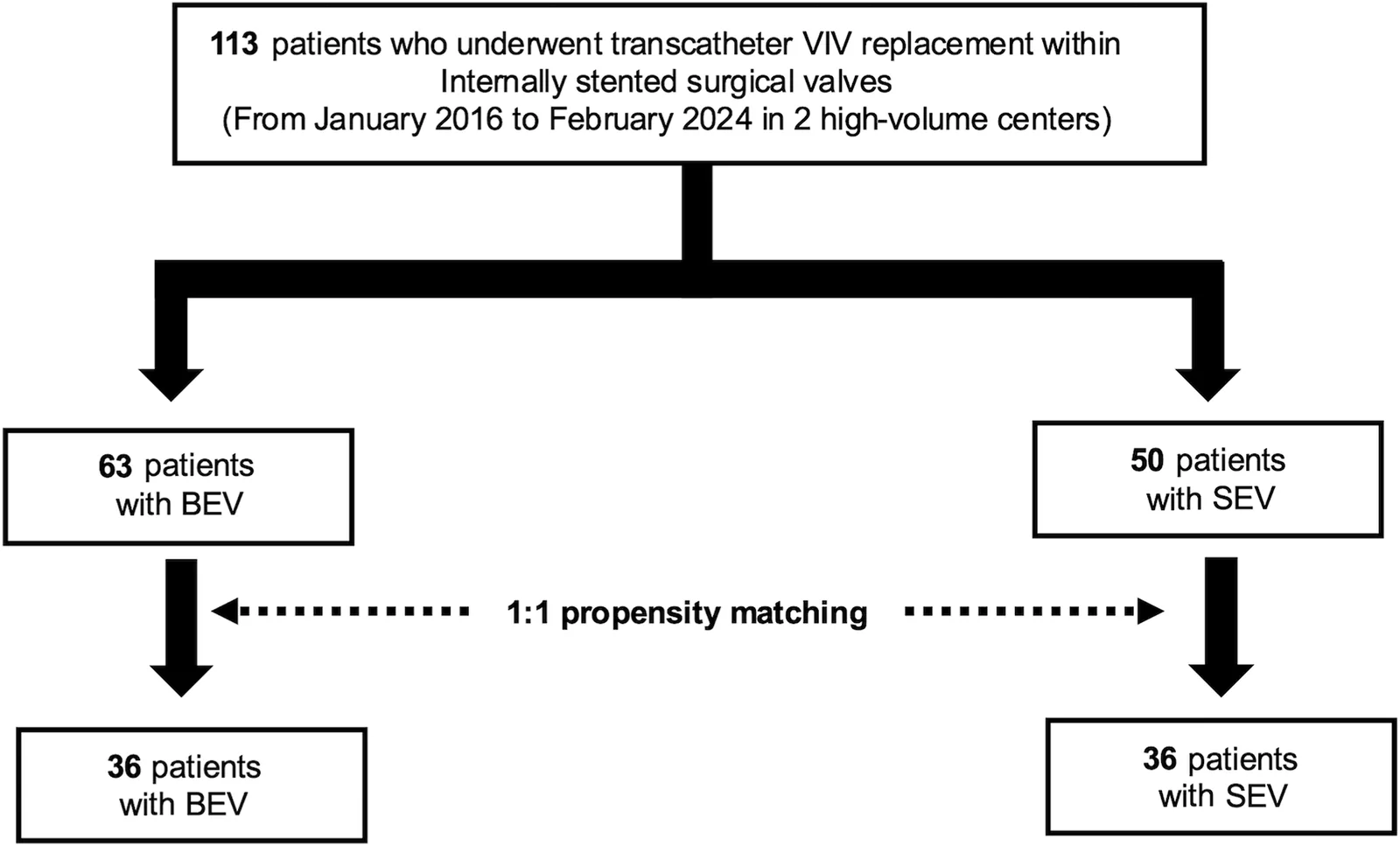
Study groups flowchart. BEV, balloon expandable valve; SEV, self-expanding valve; VIV, valve-in-valve.

### Procedural outcomes

The procedural outcomes are summarized in Table 2. As the results were consistent between the unmatched and matched cohorts, only the findings for the matched cohorts are presented. Most patients underwent TAVI via a transfemoral approach. Coronary protection against potential coronary obstruction and required chimney stenting deployment were similar between the 2 groups (13.9% in the BEV group vs 11.1% in the SEV group; *P* = 1.000, and 11.1% in the BEV group vs 11.1% in the SEV group; *P* = 1.000, respectively). No patients underwent the bioprosthetic aortic scallop intentional laceration to prevent iatrogenic coronary artery obstruction (BASILICA) technique in either group. One patient in the BEV group experienced acute coronary obstruction owing to sinus sequestration (Supplemental Fig. S1). In the SEV group, post-dilatation was performed more frequently than it was in the BEV group (25.0% vs 2.8%; *P* = 0.014). The groups had no significant differences in the mean pressure gradient after TAVI. No patients in either group had moderate or severe aortic regurgitation (AR). The incidence of mild AR was higher in the SEV group than in the BEV group (5.6% vs 17.1%).

**Table 2:** Procedural results

Variables	Unmatched cohort			Matched cohort		
	BEV (n = 63)	SEV (n = 50)	*P*	BEV (n = 36)	SEV (n = 36)	*P*
**Procedure data**						
Vascular access			**0.019**			0.239
Transfemoral	55 (87.3)	49 (98.0)		33 (91.7)	36 (100)	
Transapical	8 (4.5)	0		3 (8.3)	0 (0)	
Transaxillary	0	1 (2.0)		0 (0)	0 (0)	
Prosthesis size, mm			**<0.001**			**<0.001**
20	20 (31.7)	-		13 (36.1)	-	
23	39 (61.9)	41 (82.0)		21 (58.3)	27 (75.0)	
26	4 (6.3)	7 (14.0)		2 (5.6)	7 (19.4)	
29	0	2 (4.0)		0	2 (5.6)	
Coronary protection for potential coronary occlusion	10 (15.9)	7 (14.0)	0.782	5 (13.9)	4 (11.1)	1.000
Single	6 (9.5)	3 (6.0)		3 (3.2)	2 (5.6)	
Both	4 (6.3)	4 (8.0)		2 (5.6)	2 (5.6)	
Required chimney stenting deployment	4 (6.3)	7 (14.0)	0.211	4 (11.1)	4 (11.1)	1.000
Successful valve implantation	63 (100)	50 (100)	1.000	36 (100)	36 (100)	1.000
Conversion to surgery	0 (0)	0 (0)		0 (0)	0 (0)	
Pre-dilatation	1 (1.6)	3 (6.0)	0.320	0 (0)	2 (5.6)	0.493
Post-dilatation	1 (1.6)	10 (20.0)	**0.002**	1 (2.8)	9 (25.0)	**0.014**
**Echocardiography after TAVI (before discharge)**						
LVEF, %	48.8 ± 9.8	51.4 ± 10.7	0.748	48.3 ± 9.2	51.8 ± 10.3	0.596
Effective orifice area, cm^2^	1.07 ± 0.24	0.97 ± 0.27	0.418	1.03 ± 0.24	1.07 ± 0.31	0.406
AVmax, m/s	3.1 ± 0.6	2.7 ± 0.6	0.521	3.1 ± 0.6	2.6 ± 0.6	0.826
Mean aortic gradient, mm Hg	23.0 ± 8.9	20.7 ± 11.1	0.100	23.2 ± 9.7	18.1 ± 9.2	0.637
Mean aortic gradient ≥ 20 mm Hg	34 (54.8)	24 (48.0)	0.472	19 (52.8)	14 (38.9)	0.237
Aortic regurgitation			0.209			0.151
None/trace	58 (93.5)	42 (85.7)		34 (94.4)	29 (82.9)	
Mild	4 (6.5)	7 (14.3)		2 (5.6)	6 (17.1)	
Moderate/severe	0 (0)	0 (0)		0 (0)	0 (0)	

Values are expressed as total number (percentage), mean ± standard deviation, or median (interquartile range). Boldface indicates significance.

AVmax, aortic valve maximum blood flow velocity; BEV, balloon-expandable valve; LVEF, left ventricular ejection fraction; SEV, self-expanding valve; TAVI, transcatheter aortic valve implantation.

– indicates a valve size not available for that device (Evolut is not manufactured in 20 mm).

### Clinical outcomes and follow-up results

Regarding the 30-day clinical outcomes, the 2 groups had no significant differences in the incidence of adverse events, including death, stroke, permanent pacemaker implantation, acute kidney injury, major vascular complications, and major or life-threatening bleeding (Table 3).

**Table 3:** Follow-up data

Outcomes	Unmatched cohort			Matched cohort		
	BEV (n = 63)	SEV (n = 50)	*P*	BEV (n = 36)	SEV (n = 36)	*P*
**At 30-d follow-up**						
All-cause of death	2 (3.2)	1 (2.0)	1.000	1 (2.8)	0 (0)	1.000
Cardiovascular death	2 (3.2)	1 (2.0)	1.000	1 (2.8)	0 (0)	1.000
Stroke	0 (0)	0 (0)		0 (0)	0 (0)	
Acute coronary artery occlusion	1 (1.6)	0 (0)	1.000	1 (2.8)	0 (0)	1.000
Permanent pacemaker∗	1 (1.6)	2 (3.2)	0.583	0 (0)	2 (5.6)	0.493
Acute kidney injury	2 (3.2)	2 (3.2)	1.000	1 (2.8)	1 (2.8)	1.000
VCD success	54 (98.2)	47 (95.9)	0.600	32 (97.0)	35 (97.2)	1.000
Major/life-threatening bleeding	9 (14.3)	6 (12.0)	0.722	4 (11.1)	4 (11.1)	1.000
Valve reintervention	0 (0)	0 (0)		0 (0)	0 (0)	
**At 1 Y**						
All cause of death	6 (9.5)	4 (8.0)	1.000	3 (8.3)	3 (8.3)	1.000
Death or heart failure hospitalization	7 (11.1)	6 (12.0)	0.883	3 (8.3)	5 (13.9)	0.710
Late coronary occlusion	0 (0)	0 (0)		0 (0)	0 (0)	
**Echocardiography at 1 Y**						
	**(n = 35 of 63)**	**(n = 32 of 50)**		**(n =18 of 36)**	**(n = 22 of 36)**	
LVEF, %	58.5 ± 9.5	60.9 ± 9.7	0.936	61.8 ± 7.0	59.9 ± 10.0	0.165
Mean aortic gradient, mm Hg	23.4 ± 9.1	13.1 ± 6.3	0.080	22.7 ± 8.4	11.7 ± 4.3	**0.020**
Mean aortic gradient ≥ 20 mm Hg	20 (58.8)	4 (12.1)	**<0.001**	11 (61.1)	1 (4.5)	**<0.001**
Aortic regurgitation			1.000			1.000
None-trace	33 (94.3)	31 (96.9)		17 (94.4)	21 (95.5)	
Mild	2 (5.7)	1 (3.1)		1 (5.6)	1 (4.5)	
Moderate/severe	0 (0)	0 (0)		0 (0)	0 (0)	
**Long-term follow-up (median 1296 d [interquartile range: 734-1833])**						
	**(n = 63)**	**(n = 50)**		**(n = 36)**	**(n = 36)**	
All cause of death	21 (33.3)	10 (20.0)	0.115	8 (22.2)	8 (22.2)	1.000
Late coronary occlusion	0 (0)	1 (2.0)	0.442	0 (0)	0 (0)	
Valve reintervention	1 (1.6)	0 (0)	1.000	0 (0)	0 (0)	

Values are expressed as total number (percentage), or mean ± standard deviation, unless otherwise indicated. Boldface indicates significance.

BEV, balloon-expandable valve; LVEF, left ventricular ejection fraction; SEV, self-expanding valve; VCD, vascular closure device.

∗ Patients with previous permanent pacemaker implementation were excluded.

The median long-term follow-up duration for the surviving patients was 1258 days (IQR: 756-1725). Clinical follow-up, including all-cause mortality and heart failure hospitalization, was obtained for all patients in the matched cohort. Echocardiographic follow-up at 1 year was available in 18 patients (50.0%) in the BEV group and 22 patients (61.1%) in the SEV group. The incidence of all-cause mortality at 2 years, and the composite endpoints of all-cause mortality, hospitalization for heart failure, or coronary obstruction at 2 years were similar between the groups (log-rank: *P* = 0.695 and *P* = 0.489, respectively; Fig. 2). The mean transvalvular gradient at 1 year was higher in the BEV group than in the SEV group (23 [IQR: 19-33] vs 11 [IQR: 7-16]; *P* < 0.001). The proportion of mean transvalvular gradient ≥ 20 mm Hg at 1-year follow-up was significantly greater in the BEV group (61.1% vs 4.5%; *P* < 0.001). No significant differences were observed in the left ventricular ejection fraction or AR between the 2 groups. In the overall cohort, one patient in the SEV group developed late chimney stent occlusion, and one patient in the BEV group required reintervention with redo TAVI (transcatheter aortic valve-in-transcatheter aortic valve-in-surgical aortic valve [TAV-in-TAV-in-SAV]), due to structural valve deterioration during follow-up.

**Figure 2:**
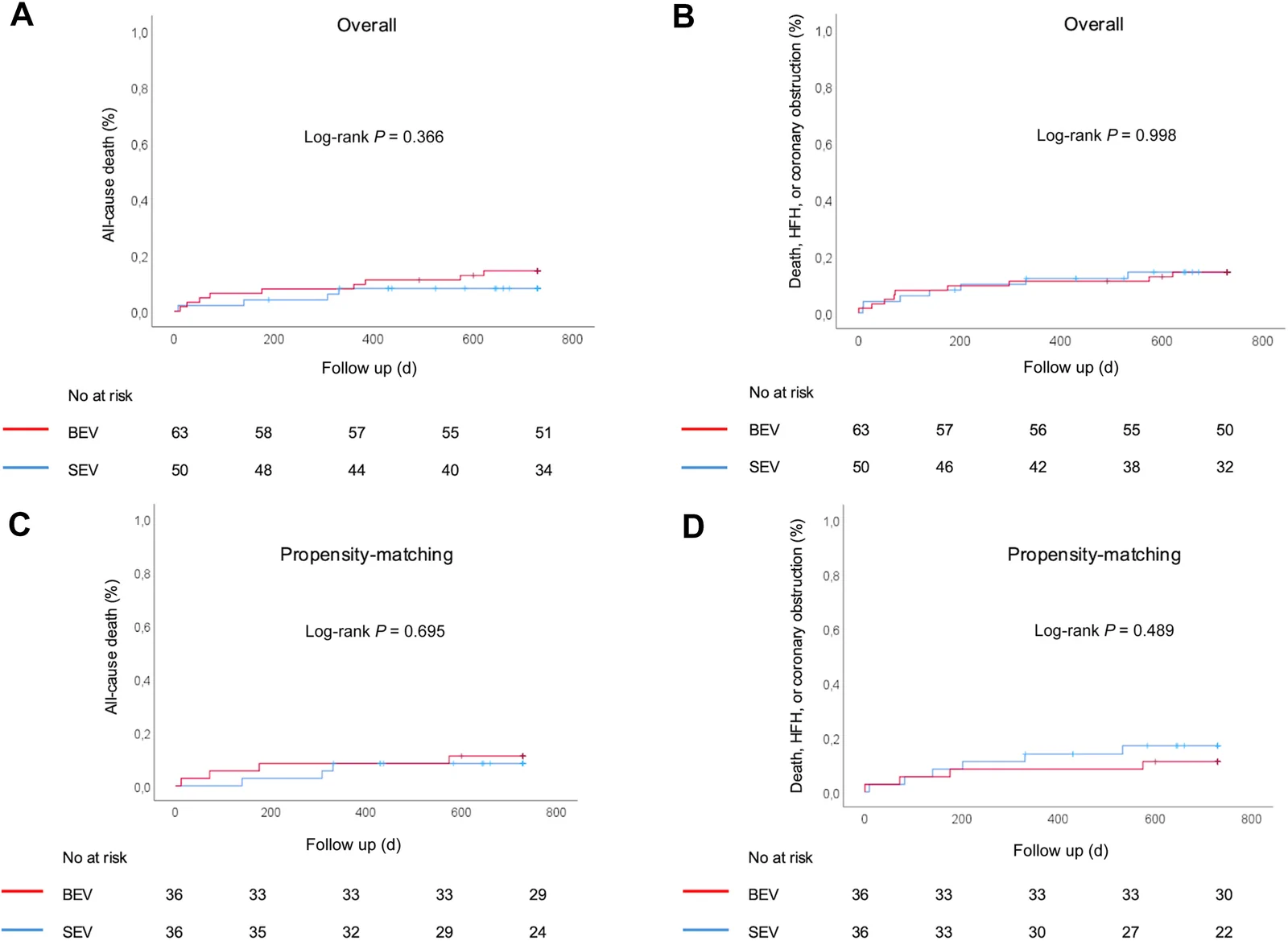
Cumulative incidence of clinical outcomes, evaluated using Kaplan-Meier analysis. There was no significant difference between the groups In (**A, B**) the overall cohort and (**C, D**) the propensity-matched cohort on the basis of age, sex, body mass index, Society of Thoracic Surgeons mortality score, hypertension, diabetes mellitus, atrial fibrillation, chronic kidney disease, left ventricular ejection fraction, chronic pulmonary disease, peripheral artery disease, or true internal diameter. BEV, balloon-expandable valve; HFH, heart failure hospitalization. SEV, self-expanding valve.

In a sensitivity analysis using multivariate Cox regression performed on the unmatched cohort, the Society of Thoracic Surgeons mortality score was independently associated with the composite endpoint of all-cause mortality, hospitalization for heart failure, and coronary obstruction at 2 years (adjusted hazard ratio: 1.08; 95% confidence interval: 1.04-1.12; *P* < 0.001; Table 4).

**Table 4:** Cox regression analysis of composite endpoint at 2 years in the unmatched cohort

Characteristic	HR (95% CI)	*P*	Adjusted HR (95% CI)	*P*
Age, per Y	1.02 (0.93–1.12)	0.648		
Female	0.52 (0.19–1.40)	0.195		
BMI	1.02 (0.92–1.12)	0.775		
LVEF, %	0.98 (0.95–1.02)	0.419		
Small valve (true ID < 19)	0.93 (0.30–2.87)	0.893		
Etiology AS	2.55 (0.89–7.33)	0.083	2.11 (0.71–6.26)	0.180
Etiology AR	0.64 (0.18–2.25)	0.489		
Etiology mixed	0.38 (0.09–1.68)	0.203		
STS score, %	1.09 (1.05–1.13)	**< 0.001**	1.08 (1.04–1.12)	**< 0.001**
BEV (vs SEV)	1.00 (0.37–2.68)	0.998		
Prior PCI	1.01 (0.23–4.42)	0.995		
Prior CABG	0.97 (0.34–2.78)	0.950		
Prior permanent pacemaker	0.04 (0.00–64.3)	0.398		
Coronary protection for potential coronary occlusion	1.88 (0.61–5.84)	0.273		
Pre-dilatation	1.94 (0.26–14.7)	0.522		
Post-dilatation	1.33 (0.30–5.88)	0.703		
Pre-mean aortic gradient	1.01 (0.99–1.04)	0.330		

AR, aortic regurgitation; AS, aortic stenosis; BEV, balloon-expandable valve; BMI, body mass index; CABG, coronary artery bypass grafting; CI, confidence interval; HR, hazard ratio; ID, internal diameter; LVEF, left ventricular ejection fraction; PCI, percutaneous coronary intervention; SEV, self-expanding valve; STS, Society of Thoracic Surgeons.

## Discussion

To the best of our knowledge, this study is the first to compare the clinical and hemodynamic outcomes of BEVs and SEVs in ViV-TAVI for IS surgical bioprostheses.

The main findings of the current analysis were as follows: in the PSM cohort, (i) early and 2-year clinical outcomes, including all-cause mortality, hospitalization for heart failure, and coronary obstruction, were comparable in patients who underwent VIV-TAVI for IS with BEV vs SEV; (ii) the SEV group had a lower post-procedural mean transvalvular gradient at the 1-year follow-up than the BEV group (Central Illustration).

**Central Illustration:**
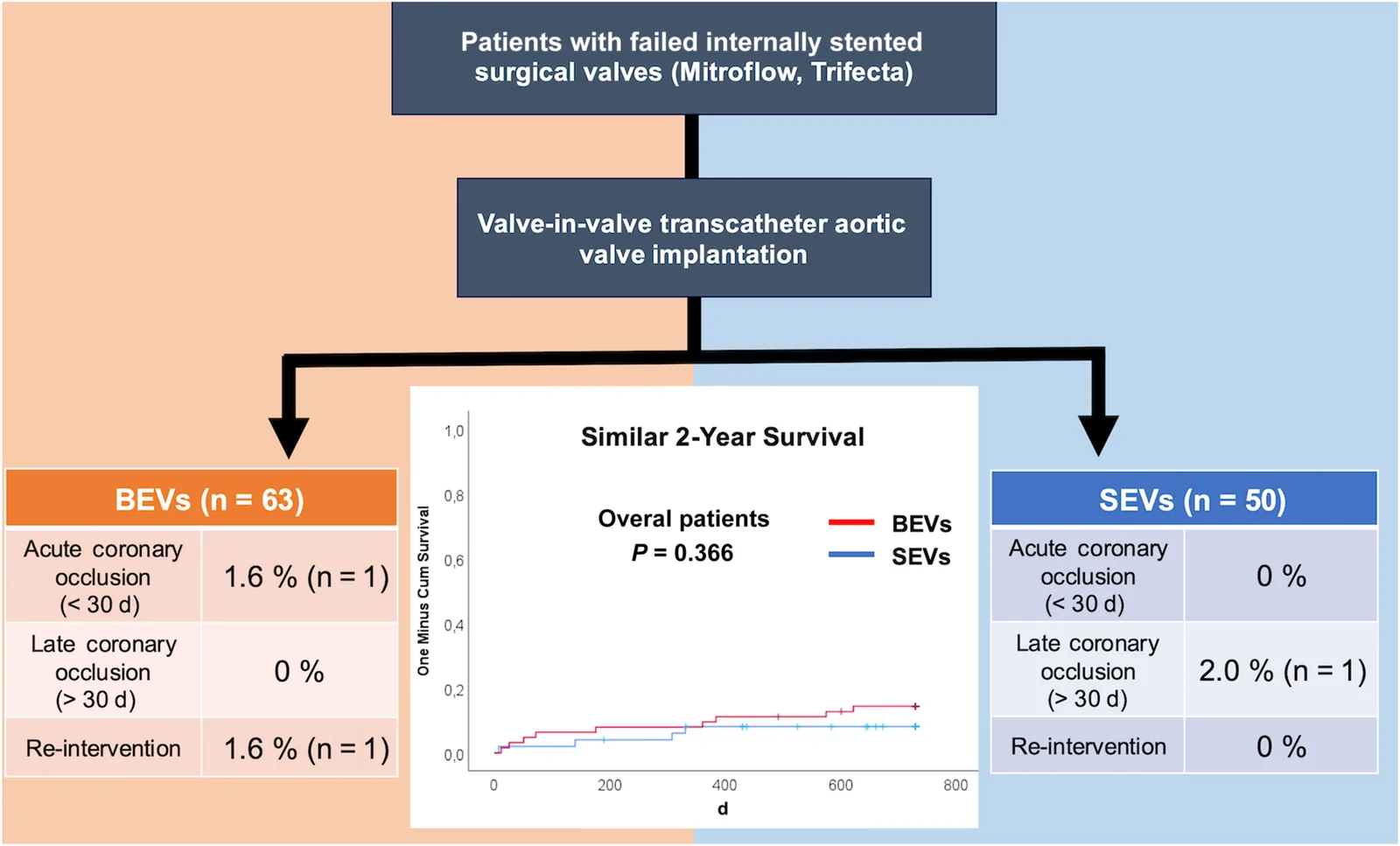
Clinical and hemodynamic outcomes for transcatheter aortic valve implantation in internally stented surgical valve: comparison of balloon-expandable valves (BEVs) and self-expanding valves (SEVs). Mitroflow is from Sorin Group (Arvada, CO) and Trifecta is from St. Jude Medical (St. Paul, MN).

### Clinical outcomes and hemodynamic status

ViV-TAVI represents an alternative treatment for repeat surgical aortic valve replacement, and the procedural volume is expected to increase. From the Valve-in-Valve International Data (VIVID) Registry, having a small bioprosthesis represented an important risk factor for mortality after the ViV-TAVI procedure over long-term follow-up.^2^ ViV-TAVI in small surgical bioprostheses has been associated with a higher risk of PPM. Moreover, preexisting PPM has an elevated risk of mortality following ViV-TAVI.^2^ In patients who underwent TAVI with native aortic stenosis, especially small annuli, the residual transvalvular echocardiographic gradient was lower, and a lower rate of moderate-to-severe PPM was achieved with an SEV, compared with a BEV.^10^, ^11^, ^12^ In our study, about 75% of the patients in the PSM cohort had a small index device (true ID ≤ 19 mm). Compared with the SEV group, the BEV group demonstrated a higher mean transaortic gradient by approximately 5 mm Hg, and had a greater proportion of patients with a mean transaortic gradient ≥ 20 mm Hg before discharge. These differences persisted at the 1-year follow-up assessment, with mean transaortic gradients 11 mm Hg higher in the BEV group than in the SEV group. These findings may be attributable to the supra-annular design of the SEV. In addition, the nominal size of the SEV is larger than that of the BEV, despite both valves being indicated for comparable ranges of surgical bioprosthesis inner diameters. This size difference between the 2 platforms also may have contributed to the hemodynamic performance.

In our cohort, post-dilatation during the TAVI procedure was performed more frequently in patients with an SEV than in those with a BEV. Optimizing THV expansion may contribute to favourable long-term hemodynamic outcomes, including lower residual gradients and a reduced risk of structural valve deterioration. For the BEV, Husain et al. demonstrated that post-dilatation, using the delivery system balloon at nominal volume was associated with improved THV expansion in patients with native aortic stenosis.^13^ The SEV platform may be more prone to underexpansion within a rigid, IS surgical valve, thereby prompting additional balloon dilatation to achieve optimal frame expansion. In addition, bioprosthetic valve fracture, which occurs when the surgical bioprosthetic valve ring fractures with a high-pressure balloon inflation, may be an option to improve hemodynamic performance, resulting in lower residual transvalvular gradient.^14^ Although bioprosthetic valve fracture has a potential risk for coronary obstruction and THV damage, our findings suggest that post-dilatation may have contributed to optimizing hemodynamic performance.

The clinical significance of a high residual gradient following TAVI remains inconsistent. Previous studies have shown that high residual transvalvular echocardiographic gradients following ViV-TAVI are not associated with clinical outcomes, including major adverse events.^15^ In the randomized LYTEN (Long-term outcomes of Young Transcatheter aortic valve replacement Evaluation and Network) trial, the supra-annular SEV was associated with lower transvalvular gradient compared to the BEV; however, no significant differences were observed in functional status, clinical outcomes, and quality of life at the 1-year follow-up assessment.^6^ In our study, despite a worse hemodynamic profile in patients with a BEV, no significant differences were observed between the 2 groups in composite outcomes of all-cause mortality, hospitalization for heart failure, or coronary obstruction. The sensitivity analysis in the unmatched cohort showed that small valve size (true ID ≤ 19 mm), THV type, and post-dilatation were not independent predictors of clinical outcomes. These findings may suggest that the valve type has limited impact on midterm or 2-year clinical outcomes in patients undergoing ViV-TAVI for IS bioprostheses.

### Coronary protection

Coronary obstruction, including delayed coronary obstruction, is a rare but life-threatening complication after ViV-TAVI.^16^ In comparison with other surgical aortic bioprostheses, ViV in IS procedures has a high risk for coronary obstruction due to the stented externally mounted leaflet bioprostheses.^3^ Compared with the SEV platform, the BEV design presents characteristics of the upper row of open cells and lower stent frame height, which makes it easier to engage coronary cannulation. In our study, one case with a BEV had acute coronary obstruction, which contributed to sinus sequestration, whereas one patient with an SEV developed late chimney stent occlusion during the follow-up period. Despite these events, procedural and midterm outcomes were similar for the BEV and SEV, with comparable 2-year composite outcomes.

Generally, 2 principal strategies have been established to prevent coronary obstruction during ViV procedures. The first is the chimney/snorkel stenting technique, in which a coronary stent is positioned within the coronary artery and deployed at the coronary ostium with slight protrusion into the aorta following THV implantation, particularly if coronary flow compromise is anticipated. The other is the BASILICA technique, which uses transcatheter electrosurgery to intentionally lacerate the bioprosthetic leaflet.^17^ In the present study, all patients requiring coronary protection underwent the chimney stenting technique, and most of them required chimney stent implantation. Both chimney stenting and the BASILICA technique effectively prevent TAVI-induced acute coronary obstruction and demonstrate acceptable 1-year outcomes.^18^ The BASILICA technique has been introduced to overcome potential limitations of chimney stenting regarding lifetime management, such as stent distortion, thrombosis, difficulty in coronary re-access, and stent compression in case of future valve intervention.^19^ Conversely, the BASILICA technique remains a technically demanding procedure and may not be sufficient to splay the leaflet due to the bulky leaflet and perimeter calcium.^20^ Moreover, long-term follow-up data with these coronary protection techniques were limited. Recently, undermining iatrogenic coronary obstruction with a radiofrequency needle has been reported as an alternative coronary protection technique for the ViV-TAVI procedure.^21^ Further study should be conducted to establish the safety and efficacy of optimal coronary protection strategies.

### Study limitations

This study has several limitations. First, as an observational study, it is subject to inherent bias and confounding factors. In addition, valve selection was influenced by institutional device availability, with 1 centre using exclusively SEVs during the study period. Although PSM was performed to adjust for baseline differences as a covariate, the influence of residual confounding factors, including institutional practice patterns, cannot be completely ruled out. Second, the relatively small sample size may have limited the statistical power to detect differences between groups. Among the matched cohort, 1-year echocardiographic data were available in 50.0% of the BEV group and 61.1 % of the SEV group. Although clinical follow-up information, including mortality and heart failure hospitalization, was obtained through medical records or telephone interviews, echocardiographic assessment requires in-person visits. Therefore, the echocardiographic findings should be interpreted as preliminary and hypothesis-generating. Larger multicentre studies with more complete imaging follow-up assessment are warranted to confirm these findings. Third, although the echocardiographic and angiographic assessments were performed by experienced cardiologists, our study lacked a core laboratory for imaging analyses. Finally, potential underreporting and missing echocardiographic or follow-up data should be acknowledged. Echocardiographic follow-up assessment beyond 1 year was limited. Consequently, 2-year hemodynamic comparisons were not performed.

## Conclusions

In patients who underwent ViV-TAVI with the IS bioprosthesis, midterm clinical outcomes were comparable between the SEV and BEV groups, whereas patients receiving an SEV had a lower transvalvular gradient at the 1-year follow-up assessment. However, given the limited echocardiographic follow-up, these findings should be interpreted with caution. Further studies with a longer follow-up period and a larger population are necessary to provide information on the impact of the SEV and BEV on long-term clinical outcomes, prosthesis durability, and the occurrence of adverse events, including late coronary obstruction and valve reintervention.

## Ethics Statement

The study protocol conformed to the principles of the Declaration of Helsinki and was approved by the institutional clinical research and ethics committee (TGE01492-024).

## Patient Consent

The authors confirm that patient consent is not applicable to this article. This trial is the retrospective nature of the study and uses de-identified data; therefore, the requirement for informed consent was waived.

## Funding Sources

The authors have no funding sources to declare.

## Disclosures

M.J. reports receiving honoraria for lectures from Edwards Lifesciences and Medtronic. M.L. reports receiving nonregulatory research grants from Teleflex; and consultant fees from Boston Scientific Corporation, Edwards Lifesciences, and Medtronic. S.S. reports being a clinical proctor of Edwards Lifesciences (SAPIEN), Medtronic (CoreValve), and Abbott (Navitor). S.D. reports receiving honoraria for lectures from Edwards Lifesciences. T.V. reports being a clinical proctor of Edwards Lifesciences. K.S. reports receiving honoraria for lectures from Medtronic. T.O. reports receiving honoraria for lectures from Edwards Lifesciences and Medtronic. Y.S. reports receiving honoraria for lectures from Edwards Lifesciences. N.M. reports receiving honoraria for lectures from Abbott, Edwards Lifesciences, and Medtronic; and being a clinical proctor of Edwards Lifesciences and Boston Scientific Corporation. The other authors have no conflicts of interest to disclose.
