# Supplementary material for: Balloon-Expandable Vs Self-Expanding Valves for Transcatheter Aortic Valve Implantation in Internally Stented Surgical Valves
Source: CJC Open. 2026 Jun 29;8(10):1355–64. doi: 10.1016/j.cjco.2026.06.013 (PMC13654392; doi:10.1016/j.cjco.2026.06.013)
Supplement: Supplementary file 1 [file mmc1.pdf]

Supplemental Material

Supplemental Figure S1. Acute coronary obstruction due to the sinus sequestration

Supplemental Figure S1. Acute coronary obstruction due to the sinus sequestration

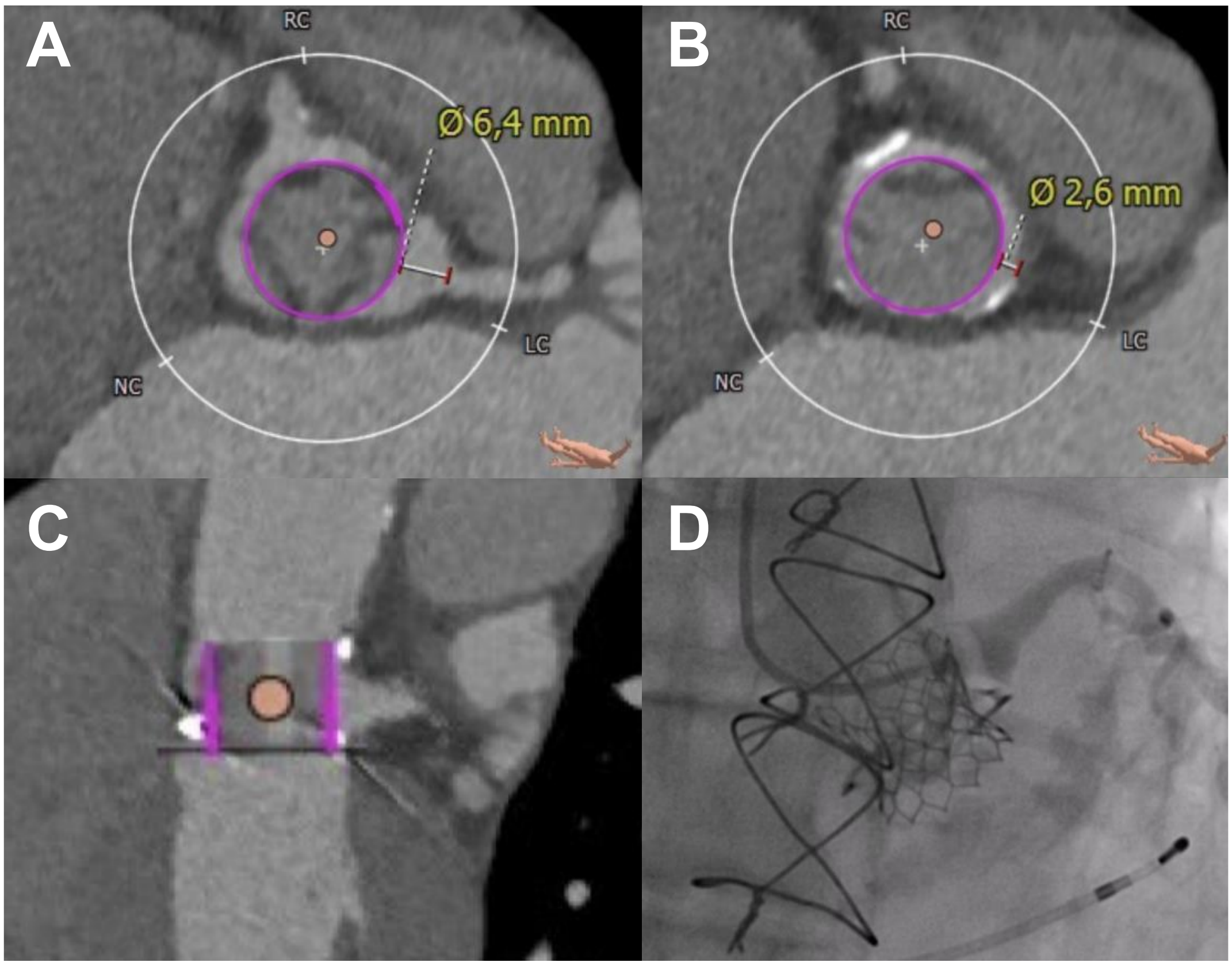

(A-C) Pre-procedural CT analysis showed that VTC-LCA was 6.4 mm, and left VTSTJ-Left was 2.6 mm. (D) After the THV implantation, electrocardiogram showed ST-segment elevation. Coronary angiography was performed, but the complication was not noticed at that time. After the procedure, troponin I was elevated and apical hypokinesia was noted in echocardiography, leading to the acute coronary obstruction due to the sinus sequestration.

CT = computed tomography, LCA = left coronary artery, STJ = sinotubular junction, THV = transcatheter heart valve, VTC = virtual THV to coronary ostium distance, VTSTJ = virtual THV to STJ distance
